# Elevational gradient and human effects on butterfly species richness in the French Alps

**DOI:** 10.1002/ece3.2803

**Published:** 2017-04-15

**Authors:** Arnaud Gallou, Yann Baillet, Gentile Francesco Ficetola, Laurence Després

**Affiliations:** ^1^ Laboratoire d'Ecologie Alpine UMR5553Université Grenoble‐Alpes, CNRSGrenobleFrance; ^2^ FlaviaA.P.E. Association pour l’Etude des PapillonsTreptFrance; ^3^ Department of BiosciencesUniversita degli Studi di MilanoMilanoItaly

**Keywords:** arable lands, elevational gradient, permanent crops, species range, species richness, urbanization

## Abstract

We examined how butterfly species richness is affected by human impact and elevation, and how species ranges are distributed along the elevational gradient (200–2700 m) in the Isère Department (French Alps). A total of 35,724 butterfly observations gathered in summer (May–September) between 1995 and 2015 were analyzed. The number of estimated species per 100‐m elevational band was fitted to the elevational gradient using a generalized additive model. Estimations were also performed on a 500 m × 500 m grid at low altitude (200–500 m) to test for the human impact on species richness using generalized least squares regression models. Each species elevational range was plotted against the elevational gradient. Butterfly richness along the elevational gradient first increased (200–500 m) to reach a maximum of 150 species at 700 m and then remained nearly constant till a sharp decrease after 1900 m, suggesting that after some temperature threshold, only few specialized species can survive. At low elevation, urbanization and arable lands had a strongly negative impact on butterfly diversity, which was buffered by a positive effect of permanent crops. Butterfly diversity is exceptionally high (185 species) in this alpine department that represents less than 5% of the French territory and yet holds more than 70% of all the Rhopalocera species recorded in France. Both climate and habitat shape the distribution of species, with a negative effect of anthropization at low altitude and strong climatic constraints at high altitude.

## 1 Introduction

Species richness distribution along elevational gradients is a topic that has intrigued ecologists and biogeographers since decades (Rohde, 1999). Multiple studies have documented species distribution along elevational and latitudinal gradients in a variety of habitats and taxa, and many mechanisms have been suggested to explain spatial variation in species richness (Hawkins et al., 2003; McCain & Grytnes, 2010; Rahbek, 2005; Szewczyk & McCain, 2016). However, the processes underlying species distribution along elevational gradients are still poorly understood. It is generally accepted that species diversity declines with increasing elevation, but such declines are rarely straightforward. Several metaanalyses conducted on various taxa have found evidence for four common species diversity patterns along elevational gradients: monotonic decrease, low plateau (consecutively high richness across the lower portion of the gradient), low plateau with a mid‐elevational peak (high richness across low elevations with a diversity maximum at mid‐elevation), and unimodal mid‐elevation peak, the latter being the most common (McCain & Grytnes, 2010; Rahbek, 2005). The mid‐domain effect predicts that the random placement of species ranges within a bounded biogeographical domain produces a peak of richness at intermediate elevations, due to geometric constraints (Colwell & Hurtt, 1994). Other hypotheses have been proposed to explain the unimodal pattern, including highest productivity at mid‐elevations (Mittelbach et al., 2001; Sanders, 2002) or interactions with human activities at lower elevations (Bharti, Sharma, Bharti, & Pfeiffer, 2013; McKinney, 2002; Nogues‐Bravo, Araujo, Romdal, & Rahbek, 2008). Elevational gradients are tightly interconnected with human activities, and both climate and local factors (e.g., land use) are likely interacting to explain the species richness patterns observed along elevational gradients.

Butterfly diversity is known to be particularly high in mountain regions, presumably because elevational gradients encompass several gradients in climatic and environmental factors (especially temperature and moisture) and vegetation assemblages vary along elevational gradients, contributing to environmental heterogeneity (Pellissier et al., 2013). Butterfly populations are also considered good indicators of environmental changes as they react more sensitively to habitat loss and decline more rapidly than birds and plants in regions with high human pressure (Thomas et al., 2004). Human effects on butterfly diversity can be negative through urbanization (habitat loss) and habitat conversion to arable lands but, on another hand, human activities such as traditional agriculture maintain some open habitats (such as permanent croplands or extensive pastures) and promote environmental heterogeneity (Uchida, Hiraiwa, & Ushimaru, 2016) that can represent favorable habitats for butterflies (Bartonova, Benes, Fric, Chobot, & Konvicka, 2016; Botham et al., 2015; Horak & Safarova, 2015; Jew, Loos, Dougill, Sallu, & Benton, 2015).

The aim of the present study is to analyze the pattern of elevational richness of butterflies in an area of high human pressure in the French Alps, based on a set of bioclimatic, land cover and >35,000 occurrence data of butterfly. More precisely, we asked whether butterfly richness decreases monotonically with increasing elevation or exhibits a peak at intermediate elevations. We further examined the effects of anthropization (urbanization and arable land) and other landscape characteristics (forest, permanent crops, grassland, sparse vegetation) on species richness.

## 2 Methods

### 2.1 Study area

**Figure 1:**
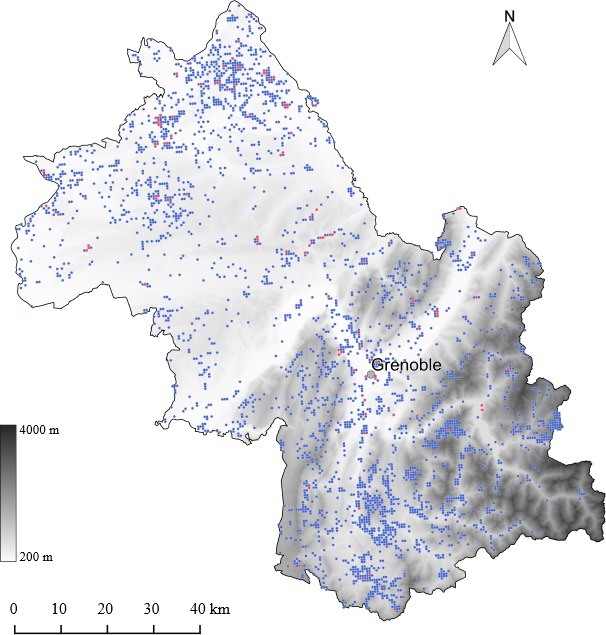
Topographic map of the Isère department and distribution of all the 4,776 sites with at least one observation (in blue) and of the 75 sites (in red) with species estimation more than 70%, of which 64 were used for analyzing the impact of man at low altitude (<500 m). The city of Grenoble (>500,000 inhabitants) is indicated

This study was carried out in the northwestern Alps, Isère department, France (Figure 1). The department of Isère extends *c*. 130 km from north to south and *c*. 120 km from east to west with a total area of 7,431 km^2^ and is densely populated (157.37 inhabitant/km^2^). The southeastern half of Isère consists in a mountainous region with four main mountain ranges, namely Chartreuse, Vercors, Belledonne and the northernmost part of the Écrins, each being geographically separated by deep valleys and with an elevational range spanning from 200 m up to >3,500 m in the Écrins (~3,000 m in Belledonne, and ~2,000 m in Vercors and Chartreuse). The northwestern half of Isère is mainly characterized by hills, most not exceeding 700 m. About half the total surface of the department lies between 200 and 500 m.

At low elevations, the landscape is mainly rural or semiurban and consists of patches of agricultural lands, villages or small towns, and deciduous forests. Human impact in this region is not new as there are many traces of human activities back to the Mesolithic in valleys (the roman cities of Vienna, Grenoble (Cularo), and Cremieux were constructed on old prehistorical settlements (Bocquet et al., 1987) and in mountains (Martin, Delhon, Thiébault, & Pelletier, 2012). Traditional agriculture and pasture have shaped the landscape since thousands of years. Urbanization and intensive agriculture (mainly corn crops) have risen dramatically during the last decades in the three main river beds (Rhône, Isère, Drac) at the expense of swamps and wetlands that constituted the main landscape elements in the bottom of these large glacier valleys, and Grenoble is now the tenth city in France with more than 500,000 inhabitants. Rural areas (small cities, traditional extensive agriculture) can reach up to *c*. 1,500 m in the southeastern part of the department. Human impact is not restricted to lowlands in Isère: forestry represents a major resource for this mountainous department since the sixteenth century, and it was the first department in France to develop hydroenergy‐based industries during the nineteenth century; finally, it is one of the most attractive touristic areas in Europe (many recreative areas including ski resorts). The intermediate elevations are dominated by mixed and coniferous forests (mostly managed) which are replaced at higher elevations (>1,700 m) by grasslands (alpine meadows, used as pastures and harboring ski resorts) and finally by bare rocks and/or glaciers (~>2,300 m). Human activities generate a mosaic of habitats more or less favorable to butterflies (urban, arable lands, pastures, permanent crops, sparse vegetation). In addition to human activities largely shaping the landscape, climatic conditions are particularly diverse in Isère. The influence of a continental, oceanic and mediterranean climate offers a wide range of environmental conditions at lower elevations which gradually turn into an alpine climate at higher elevations. For the 1995–2015 period, the mean annual temperature varies between +11 and −0.5°C, and the mean annual precipitation between 700 and 2,200 mm is depending on the elevation and the massif.

### 2.2 Butterfly data

A dataset provided by the entomological association Flavia (http://www.flavia.ape.fr) was used for investigating the relationship between butterfly species richness, niches, and environmental variables in Isère. The dataset comprised occurrence data of butterflies, including geolocalized points (exact GPS position or records at 100 m resolution), full species names (*i.e.,* family, genus, species), and the date of each observation. In order to have a recent overview of the butterfly species richness in Isère, we only considered observations performed between 1995 and 2015. Only species observed between May and September were selected and analyzed, because this corresponds to the most favorable period for butterfly activity.

The resulting dataset contains a total number of 35,724 observations from 185 species, regrouped in 71 genera in six Lepidoptera families: Hesperiidae, Lycaenidae, Nymphalidae, Papilionidae, Pieridae and Riodinidae (Table 1).

**Table 1:** Taxonomic distribution of the 185 butterfly species observed in Isère between May and September from 1995 to 2015

Family	Genus	Species
Nymphalidae	31	87
Lycaenidae	20	50
Pieridae	8	20
Hesperiidae	8	22
Papilionidae	3	5
Riodinidae	1	1
Total	71	185

### 2.3 Species richness estimation and study design

To estimate the number of species at a given site, and to link it with land cover characteristics, the department of Isère was divided into 500 × 500 m cells, and each observation falling into a cell was attributed to this site: the 35,724 observations corresponded to a total of 6,447 sites distributed across the department (Figure 1). This dataset was strongly biased toward low elevation observations: most of the sites that were sampled several times were located below 500 m. We therefore performed two types of analyses of species richness. First, we evaluated variation of species richness along the 200–2,700 m gradient by dividing it in twenty‐six 100‐m elevational bands, and we analyzed the relationships between species richness per elevational band and environmental parameters (altitude, climate, main vegetation type). Second, we focused on human impact at lower altitude (200–500 m) on species richness in 500 × 500 m grid cells.

The number of observed species in a given area is always lower than the real number of species because of undetected species, and several unbiased estimators of species richness have been developed (Gotelli & Colwell, 2011). The estimated number of species at a site relies on sampling intensity, which in turn depends on multiple variables such as accessibility, proximity to urban areas, perceived interest for researchers, or presence of research institutions (Ficetola, Bonardi, Sindaco, & Padoa‐Schioppa, 2013; Stolar & Nielsen, 2015; Yang, Ma, & Kreft, 2014). Comparative analyses showed that the first‐order jackknife is one of the best performing approaches for biodiversity estimates (Chazdon, Colwell, Denslow, & Guariguata, 1998). Species richness in each elevational band of 100 m (broad scale analysis) and in each grid cell (low elevation analysis) was estimated on the basis of occurrence data using the first‐order jackknife estimator (Colwell & Coddington, 1994), as implemented in the *“vegan”* package (Dixon, 2003) in R (R Development Core Team, 2016).

### 2.4 Environmental data

To assess the potential role of environmental variables in shaping butterfly species distribution along elevational gradient, 14 environmental predictors were selected, representing topographic, climatic, and land cover variables. Altitude was extracted from the 25‐m resolution digital elevation model from the French National Geographic institute (http://www.professionnels.ign.fr). The climatic variables were mean annual temperature, temperature seasonality (standard deviation of monthly average temperature), temperature annual range (difference between maximum and minimum temperatures of the year), mean annual precipitation, precipitation of wettest month and precipitation of driest month, and were downloaded from WorldClim (http://www.worldclim.org), downscaled at a 500 m resolution. The land cover variables were extracted from the CORINE Land Cover (CLC) Map of France (http://www.eea.europa.eu): urban areas (artificial areas, level 1, CLC classification), arable lands and other crops (agriculture, level 2 CLC), deciduous forest, mixed forest, coniferous forest, sparse vegetation, heath, natural grasslands and rock or ice (forests and seminatural areas, level 3 CLC). In order to determine how these environmental features (altitude, climatic parameters, and land cover variables) covary, we performed a principal component analysis.

The estimated number of species was reported in each elevational band of 100 m along the gradient. Generalized additive models (GAM) from the *“mgcv”* (Wood, 2011) package in R (R Development Core Team, 2016) were used to represent (1) the elevational butterfly richness pattern and (2) the variation in land cover features (surface in km^2^ per 100‐m elevational bands) along the elevational gradient. We also tested for a species richness–area relationship across elevational bands (Pearson's correlation).

### 2.5 Human impact on butterfly richness at low elevation (200–500 m)

Estimators of species richness provide more accurate results if the observed species richness is not too far from the estimated one (Chazdon et al., 1998; Colwell & Coddington, 1994). We only considered cells for which the observed species richness was at least 70% of the estimated one (jackknife first‐order estimator) and that were visited more than four times: of 6,447 sites, only 75 sites met these requirements, of which 64 were at elevation between 200 and 500 m. Therefore, the analysis of relationships between butterfly richness and landscape features was performed on 64 sites (500 × 500 m cells). A recent review has identified the most favorable management practices for European butterflies conservation (rotational mowing, extensive grazing, maintenance of seminatural open habitats at lower altitude, inside the timberline) from those that are detrimental (afforestation, draining, intensive cultures, and forestry) (Bubova, Vrabec, Kulma, & Nowicki, 2015). We therefore distinguished habitats that are defavorable to butterflies such as urban areas and arable lands because they lack nectar resources and do not allow caterpillar overwintering, from habitats more likely to be favorable for butterfly life cycle such as forests, permanent crops (pastures and orchards) and sparse vegetation (Nieto‐Sanchez, Gutierrez, & Wilson, 2015). Because the CORINE Land Cover Map was not precise enough at fine scale, in each of the grid cells analyzed below 500 m, we refined the CORINE Land Cover layers on the basis of aerial photographs in ArcMap 10.3.1 (http://www.esri.com) and defined the following categories: urban, arable lands, permanent crops (including orchards and vineyards), sparse vegetation (isolated trees, hedges), grasslands, water, swamps, and deciduous forests. Those high‐resolution land cover data were used to investigate the effect of land use on butterfly species richness at lower elevations.

We used generalized least squares (GLS) to assess the effect of land use on butterfly species richness at lower elevations (200–500 m) while taking into account spatial structure. GLS allows the incorporation of spatial structure into the error of the model and is considered among the techniques with the best performance for the analysis of spatial data (Beale, Lennon, Yearsley, Brewer, & Elston, 2010; Dormann et al., 2007). We built GLS models considering all the possible combinations of independent variables; we then ranked models on the basis of their Akaike's information criterion corrected for small sample size (AICc); the models with lowest AICc values are considered to be the “best models”. AICc may select overly complex models; therefore, we considered a complex model as a candidate model only if it had AICc less than the AICc of all its simpler nested models (Richards, Whittingham, & Stephens, 2011). For each candidate model, we calculated the Akaike's weight *w* (AICc weight), which represents the probability of the different models given the data (Lukacs et al., 2007). We also calculated the relative importance of variables (RI) by summing the AICc weights of models in which each variable is included (Wagenmakers, 2003). The variance inflation factor of all the best AICc models was <2, indicating lack of collinearity issues (Dormann et al., 2013). We used likelihood ratio *R* ^2^ ($R_{\mathrm{LR}}^{2}$) as a measure of the variance explained by the model.

### 2.6 Niche separation

For each species with more than 50 total observations, the mean altitude and standard deviation (*SD*) was calculated based on all observations. The standard deviation was taken as a proxy for niche width: species with large standard deviation have a broad elevational niche (generalists), and species with small standard deviation are restricted to a small altitude interval (specialists). All statistical analyses were performed in R 3.3.0 (R Development Core Team, 2016).

## 3 Results

**Figure 2:**
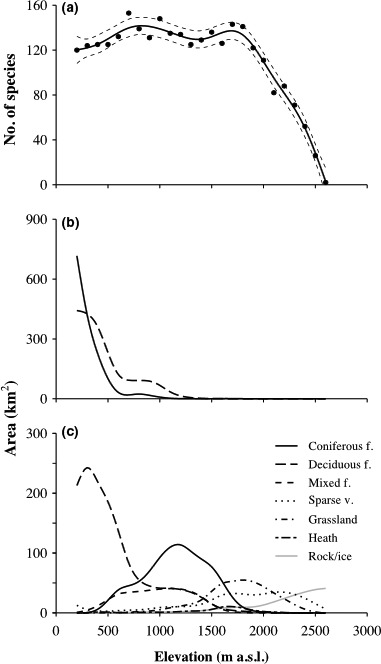
(a) Butterfly species richness variation (number of species) with elevation (m). Dots represent the number of estimated species per elevational band of 100 m, from 200 to 2,700 m. The solid line represents the response shape of the additive model and the dashed lines indicate 95% confidence interval, (b) anthropized areas (in km^2^) defined as the sum of urban and arable lands (solid line) and permanent crops (dashed line), and (c) all other habitats areas (km^2^) along the elevational gradient

The observed species richness was >60% of estimated species richness in every elevational band, with small standard errors, indicating a robust estimation (Table 2). A generalized additive model assessing the relationship between altitude and richness explained 97.5% of the deviance richness and showed AIC values much lower than a linear model (178 vs. 241), suggesting that the elevational pattern of richness was not linear. The number of butterfly species increases (gaining *c*. 30 species) in the first 500 m, peaking at 700 m with more than 150 species estimated, then remains almost constant between 800 and 1,900 m with an insignificant loss of species at *c*. 1,400 m, and finally decreases drastically from 2,000 m upwards (Figure 2a). There was no correlation between species richness and area per 100‐m elevational band (*r* = .28, *p *>* *.05).

**Table 2:** Results of the jackknife estimator on butterfly species richness per elevational bands of 100 m

Elevation (m)	Observed species	Estimated species	Standard Error	No. of visits	Species saturation
200	112	120	3	100	0.93
300	111	124	4	102	0.90
400	110	125	5	57	0.88
500	102	125	7	47	0.82
600	109	132	10	33	0.83
700	129	153	8	50	0.84
800	116	139	8	34	0.83
900	108	131	7	40	0.82
1,000	116	148	12	26	0.78
1,100	111	135	8	34	0.82
1,200	104	134	10	41	0.78
1,300	101	125	8	29	0.81
1,400	96	129	11	27	0.74
1,500	113	136	8	28	0.83
1,600	108	126	6	32	0.86
1,700	106	143	11	28	0.74
1,800	107	141	13	24	0.76
1,900	90	122	11	22	0.74
2,000	76	111	15	18	0.68
2,100	60	82	12	12	0.73
2,200	60	88	16	11	0.68
2,300	46	70	13	8	0.66
2,400	33	52	12	5	0.63
2,500	16	26	12	3	0.62
2,600	2	2	0	3	1

The two first components of the PCA performed on environmental variables explained 38% and 11%, respectively, of the variation. PC1 mainly represented a gradient of elevation and temperature, while PC2 mainly represented a gradient of land cover, ranging from areas dominated by coniferous forests to areas dominated by deciduous forests (Figure S1). The annual mean temperature was strongly correlated with elevation (*r* = .96) and decreased linearly by 0.3°C per 100 m of elevation.

Anthropized areas (urban and arable lands) are mostly found in the first 500 m where they decrease monotonically with increasing elevation and represent the largest land cover area between 200 and *c*. 500 m (Figure 2b). From *c*. 500 to *c*. 1,600 m, forest is the predominant habitat, whereas from *c*. 1,600 to *c*. 2,400 m, open areas (grasslands and heath) are dominant. Above that altitude, the landscape is dominated by bare rocks and/or glaciers (Figure 2c).

### 3.1 Relationships between human impact and butterfly richness at low altitude

**Figure 3:**
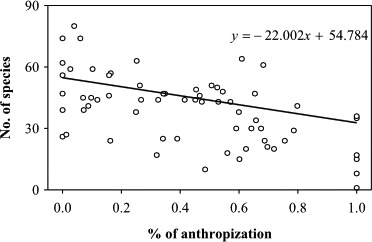
Relationship between species richness and proportion of anthropized area (urban + arable lands) in 64 sites between 200 and 500 m

Between 200 and 500 m, most of the available area was occupied by arable lands (intensive agriculture, 33%) and by deciduous forests (28%), while urban areas represented 9% of the total area. Grasslands, wetlands and swamps, remains of the most natural lowland habitats, represented less than 5% of the total area; the landscape was also constituted of pastures and orchards (permanent crops, 13%) or sparse vegetation (tree hedges, gloves, isolated trees; 10%) maintained by traditional agriculture practices. A total of 64 models were compared that tested all combinations of six variables: urban, arable, permanent crops, deciduous forest, grasslands, and sparse vegetation; wetlands and swamps were not included because the sum of all land cover variables is constrained to 1. The best AIC model included three variables: species richness was negatively related to the presence of urban and arable lands, while the relationship between species richness and permanent croplands was positive (Tables 3 and 4), with relative importance of 0.97, 0.98 and 0.78, for urban, arable, and permanent crops, respectively. The number of species decreased substantially with increasing anthropization (urban + arable lands), with 2.2 species lost every 10% more anthropized area (Figure 3).

**Table 3:** Results of the generalized least squares models testing the effect of land cover (urban, arable lands, permanent crops, sparse vegetation, grassland, and deciduous forest) on butterfly species richness between 200 and 500 m (64 sites). Percentage of each land cover variable tested was arcsin‐square‐root‐transformed. +: the variable was included in the model with a positive coefficient; −: the variable was included with a negative coefficient. AICc: Akaike's information criterion; *w*: Akaike's weight, *R* ^2^: likelihood ratio *R* ^2^. Models are ranked according to their AICc, and only models with *W *>* *0.005 are shown. A total of 64 models were tested

Arable	Deciduous	Grassland	p_crop	sparse_v	Urban	*df*	AICc	ΔAICc	*w*	*R* ^2^
−			+		−	6	523.202	0	0.7754	.3961
−	−				−	6	527.746	4.544	0.0799	.3518
−					−	5	528.103	4.901	0.0669	.3229
−		+			−	6	529.916	6.714	0.0270	.3295
−				−	−	6	530.292	7.090	0.0224	.3255
	+	+	+	+		7	532.693	9.491	0.0067	.3268
−	+		+			6	533.138	9.936	0.0054	.2948
−			+	+		6	533.276	10.074	0.0050	.2933

**Table 4:** Best AICc model relating butterfly species richness to land cover variables (200–500 m elevation)

Land cover variable	*B*	*SE*	*t*	*p*
Arable	−24.2	5.0	−4.8	<.0001
Urban	−18.3	4.6	−4.0	.0002
Permanent crops	18.3	6.8	2.7	.009

### 3.2 Species separation along the elevation gradient

**Figure 4:**
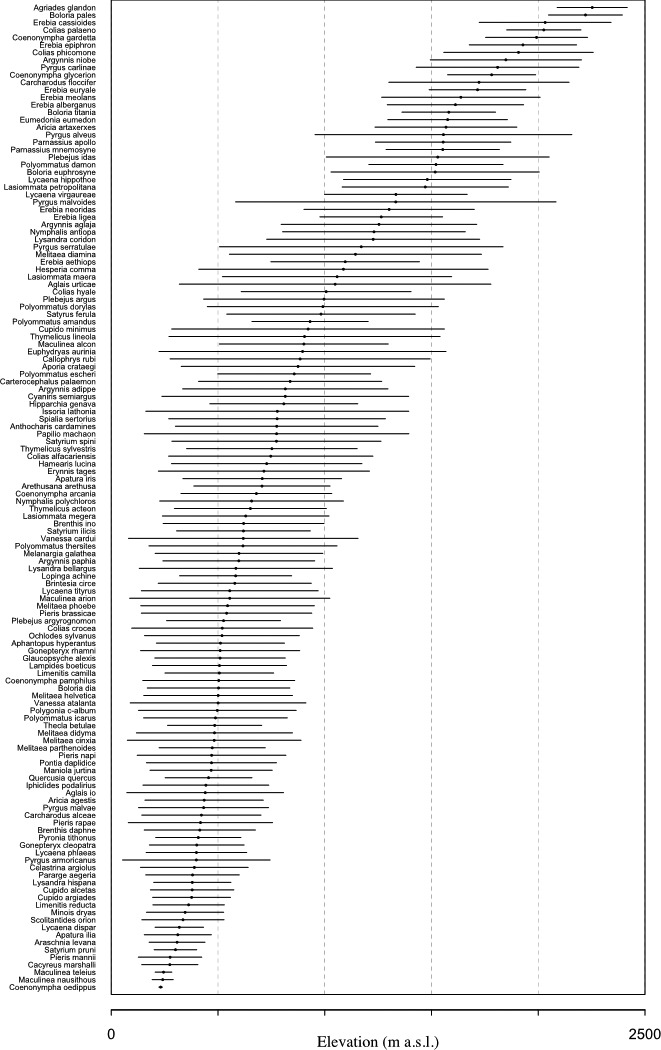
Niche width (mean altitude and standard deviation) distribution along the elevational gradient for 106 species with more than 50 observations. Dashed lines: 500‐m elevational bands

The ordination of species along elevational gradient according to their niche width showed that specialist species are restricted to the two extremes of the elevational gradient, below 500 m and above 2,000 m (Figure 4). Niche width (*SD*) ranged from 8.9 (*Coenonympha oedippus*) up to more than 750 (*Pyrgus malvoides*). Of 106 species, 56 had *SD* >350 (generalists) and 50 had *SD* <350 (specialist). There were significantly more specialist species at low (<500 m) and less specialist species at the intermediate altitude (500–1,000 m) than expected if specialists were randomly distributed across the elevational gradient (Table 5; Figure S2; Fisher exact test, odd ratio = 1.97, *p* < .05).

**Table 5:** Number of specialist species (niche breath <350) per elevational 500 m belts out of 106 species with more than 50 observations (see Figure S2)

Elevation	No of species	Niche			Number of specialists	*p*‐Value
		Median	Mean	*SD*		
0–500 m	34	242	237	105	29	.0001
500–1,000 m	45	414	437	109	8	.0008
1,000–1,500 m	13	458	491	154	3	.14
1,500–2,000 m	14	316	323	90	10	.15

## 4 Discussion

This small northwestern French Alps region is very rich in regard to butterfly diversity with 185 different species observed in Isère between 1995 and 2015, which represents more than 70% of the total number of butterfly species found in France. The butterfly species richness in Isère first increases and then decreases nonmonotonically at high elevation (>1,700 m) with no evidence of a peak of richness at mid‐elevations; indeed, a broad plateau of high species richness is observed between 700 and 1,800 m. Such mid‐elevation plateau was not previously reported as a common pattern in meta‐analyses conducted on other taxonomic groups (McCain & Grytnes, 2010). Previous studies on elevational butterfly richness patterns in the Alps reported a more straightforward decrease in richness along the elevational gradient. However, these studies examined butterfly diversity patterns at elevations higher than 600 m and in more specific habitats (Leingaertner, Krauss, & Steffan‐Dewenter, 2014; Pellissier et al., 2013). (Nogues‐Bravo et al., 2008) have emphasized the effects of sampling scale on the observed distribution patterns along altitudinal gradients, highlighting the importance of sampling along the whole elevational gradient, but very few studies are exhaustive throughout the whole gradient. Our dataset covers a large elevational range, allowing estimating with high confidence the species number in 100‐m elevational bands along the whole gradient. However, the sampling effort was not homogenous. Many more sites were visited several times at lower (<500 m) than at higher elevations. Of a total of 75 sites visited more than four times and with >70% observed/estimated species ratio, 64 were at elevations between 200 and 500 m, and only 11 at more than 500 m, thus precluding reliable estimations of species richness at the site (500 × 500 m) scale at elevations above 500 m, and it was not possible to test the landscape features leading to a mid‐elevation plateau effect. Variation in sampling intensity may generate spurious patterns in species richness along elevation gradients; that is, variation in richness may simply reflect variation in sampling intensity along this elevational gradient (Lomolino, 2001). The correlation between sampling effort and species richness was low (*r *=* *.5, *p *=* *.01), and the most intensively sampled zone (the 200–500 m elevation belt) was not the most species‐rich area, suggesting that variation in sampling effort was not a main predictor of the pattern. The species richness was not correlated to the area (*r *=* *.28, *p *>* *.05), and although the 200–500 m belt represented more than 50% of the Isère department surface, it was not the most species‐rich part of the elevation gradient. The maximum species richness was found at intermediate elevations (700–1,700 m). Many more species ranges overlap at intermediate than at extreme elevations (Figure 3), as expected by geometric constraints (mid‐domain effect); we found a significant lack of specialist species at intermediate elevations that might explain the plateau observed instead of the expected mid‐elevation richness peak. The 700–1,700 m plateau of high butterfly richness corresponds to the mixed and coniferous forest stage. Although at this altitude forest is the main landscape element, it does not constitute a continuous cover as trees are harvested (forest management, recreational areas, pastures), generating a mosaic of habitats favorable to butterflies (Viljur & Teder, 2016). Interestingly, although nonsignificant, the increase in species richness observed at ~700 and ~1,700 m (153 and 143 estimated species, respectively, Table 2) corresponds to two ecotones: the transition between the foothill and montane zones (from deciduous to coniferous forests), and the montane to subalpine zones (tree line: transition from coniferous forest to grasslands). This “ecotone effect” where species assemblages from two different bioclimatic zones meet is an acknowledged factor of species richness (Lomolino, 2001; McCain & Grytnes, 2010).

The decrease in species richness in stressful climatic conditions, particularly low temperatures, is a widely accepted explanation of the species loss at high altitudes. However, the sudden and strong decrease in butterfly richness above 1,900 m suggests a threshold environmental variable, presumably temperature, above which most butterfly species fail to complete their cycle. In addition, changes in habitat types are presumably also involved in the observed decrease, as forests are limited to below 1,800 m, and above 2,000 m there is overall decline of all the land cover categories representing natural vegetation. Actually, bare rocks become the dominant land cover category from 2,300 m upwards. All butterfly caterpillars rely on plants for their development, and decreasing butterfly richness with decreasing plant diversity is expected (Gutierrez, Vila, & Wilson, 2016). Another hypothesis is that the decrease in butterfly species richness at higher elevations might be correlated to the distance to forest edges. The fact that the species richness of butterfly is maximum at elevations below the tree line supports this hypothesis and is consistent with other studies which found that the species diversity of butterflies was higher near mixed coniferous and broad‐leaved forests (van Halder, Barbaro, Corcket, & Jactel, 2008; Luoto, Kuussaari, Rita, Salminen, & von Bonsdorff, 2001).

This study demonstrated a negative relationship between anthropization and butterfly richness between 200 and 500 m, where anthropized zones currently represent the largest part of available area. Urbanization and arable lands both had similarly strong negative effects (same relative importance of these two variables). This result is consistent with other studies that investigated the impact of human disturbances on butterfly diversity along urbanized gradients, which found that species‐poor sites were correlated with high level of urbanization (Blair, 1999; Clark, Reed, & Chew, 2007) (Lizee, Tatoni, & Deschamps‐Cottin, 2016), due to natural habitat loss produced by urban development (McKinney, 2002) (Bergerot, Fontaine, Julliard, & Baguette, 2011) and/or modern agricultural practices (Habel et al., 2016; Stefanescu, Herrando, & Paramo, 2004; Thomas, 2016). The conversion of wetlands and swamps into urban and intensive agricultural areas has been especially rapid in the Isère department, with the expansion of cities and corn crops in the large valley bottoms in only a few decades. As a result, the three species with the most restricted niche (*Coenonympha oedippus*,* Maculinea teleius,* and *M. nausithous*) are not high altitude specialists but wetland species that were more common in Isère only a few decades ago and have a large European distribution, but are now restricted to the few remaining protected areas, with little migration between them. These remnant populations are at high extinction risk due to stochastic demographic events and inbreeding (Thomas, 2016), and their situation is similar throughout their distribution range where they face the same habitat loss threat (Celik et al., 2015; Gao, Li, Chen, Guo, & Settele, 2016; van Halder et al., 2008; Jubete & Roman, 2016; Orvossy, Korosi, Batary, Vozar, & Peregovits, 2013). However, this negative effect of anthropization was buffered by a positive effect of permanent crops (extensive pastures, orchards) on butterfly richness. The amount of forest edges and clearings, as well as small‐scale agricultural mosaics of fields and forests, were found in previous studies to be the most important variables for butterfly diversity (Kivinen, Luoto, Kuussaari, & Saarinen, 2007), possibly due to high oviposition rate and high survival of larvae in those areas (Luoto et al., 2001), in addition to providing nectar resources, efficient sheltered areas to wind and refugees from predators to imagos. At a very local scale, several recent studies have emphasized the role of gardens and urban parks to buffer the negative effect of urbanization on butterflies by providing nectar resources (Fontaine, Bergerot, Le Viol, & Julliard, 2016; Lizee et al., 2016) (Sing, Dong, Wang, & Wilson, 2016). Maintaining sparsely vegetated and semiopen woodlands with glades that constitute important butterfly habitats is a recommended management strategy for conservation goals (Bubova et al., 2015; Nilsson, Franzen, & Pettersson, 2013).

## Conflict of Interest

None declared.
